# Genetic variations in the fusion protein of respiratory syncytial virus isolated from children hospitalized with community-acquired pneumonia in China

**DOI:** 10.1038/s41598-018-22826-4

**Published:** 2018-03-14

**Authors:** Xiangpeng Chen, Baoping Xu, Jiayun Guo, Changchong Li, Shuhua An, Yunlian Zhou, Aihuan Chen, Li Deng, Zhou Fu, Yun Zhu, Chunyan Liu, Lili Xu, Wei Wang, Kunling Shen, Zhengde Xie

**Affiliations:** 1 Beijing Key Laboratory of Pediatric Respiratory Infectious Diseases, Beijing Pediatric Research Institute, Beijing, 100045 China; 2 Key Laboratory of Major Diseases in Children, Ministry of Education, National Clinical Research Center for Respiratory Diseases, Beijing Children’s Hospital, Capital Medical University, National Center for Children’s Health, Beijing, 10045 China; 3 The 2nd Affiliated Hospital and Yuying Children’s Hospital of Wenzhou Medical University, Wenzhou, China; 4 Children’s Hospital of Hebei Province, Shijiazhuang, China; 5 The Children’s Hospital of Zhejiang University School of Medicine, Hangzhou, China; 6 The First Affiliated Hospital of Guangzhou Medical University, Guangzhou, China; 7 Guangzhou Women and Children’s Medical Center, Guangzhou, China; 8 Children’s Hospital of Chongqing Medical University, Chongqing, China

## Abstract

To identify the variations in fusion (F) protein gene of RSV in China, a molecular epidemiological study was conducted. A total of 553 RSV positive specimens were collected from 2338 pediatric patients hospitalized with community-acquired pneumonia during a multi-center study conducted during 2014–2016. A total of 252 samples (183 RSV A, 69 RSV B) were selected for *F* gene sequencing, and analyzed together with 142 *F* gene sequences downloaded from GenBank. The result showed that all the Chinese RSV A and RSV B strains could be divided respectively into three branches. Compared with RSV A/B prototype sequences respectively, there were significant amino acid (AA) mutations at multiple antigenic sites. For RSV A, changes were found at AA residues 122, 124, 125, 276 and 384, and for RSV B at AA residues 45, 116, 125, 172, 173 and 202. Variations in human histocompatibility leukocyte antigen-restricted CTL epitopes were also observed. In total, 56 amino acid differences for the complete F protein were found between the RSV A and B groups in China, while several mutations were only found in the RSV B strains during 2015–2016. The RSV *F* gene is relatively conserved in China, however, limited mutations are still occurring with time.

## Introduction

Human respiratory syncytial virus (RSV) is a leading pathogen of acute lower respiratory tract infection (ALRTI) among young children^1,2^. RSV caused about 33.8 million new episodes of ALRTI worldwide in children younger than 5 years of age in 2005, which contained at least 3.4 (2.8–4.3) million severe cases necessitating hospital admission. Among these patients, there were roughly 66,000–199,000 deaths, with 99% of them occurring in developing countries^2^. RSV associated illness is also the major cause of hospital admissions and mortality in infants born prematurely or with particular congenital diseases^3^. In China, RSV was also the most frequently detected virus (9.9%) in children younger than 2 years of age with ALRTI, accounting for 17.0%^4^.

Except for the therapeutic monoclonal antibody-palivizumab (MedImmune, Gaithersburg, MD, USA)^5^, there is no safe and effective vaccine or drugs against RSV available. A suitable vaccine available in the future could have a large impact in public health.

The RSV fusion (F) protein is an important transmembrane glycoprotein which mediates the fusion between virus and the target cell membrane^6^. It contains the main antigenic determinants associated with neutralizing antibodies and cytotoxic T-lymphocyte (CTL)-mediated immunity^7–9^. Antigenic sites I, II, IV, ∅, MPE-8 (AM14), α2α3β3β4 and p27 are all present in the F protein^8^. Antigenic site II is the targeted site of palivizumab with the defined binding epitope (residues 262–276)^9^. Many human histocompatibility leukocyte antigen (HLA)-restricted CTL epitopes on the F protein have been identified, such as HLA-A*01, HLA-B*57, HLA-Cw*12^10–12^. The F protein of RSV is the major candidate of vaccine research and the focus of antiviral drug or antibodies development.

It is important to elucidate variations in these epitopes of F protein. However, there has been no multi-center study of RSV F protein in China to date. There were few data of RSV F molecular variations available, especially the binding site of palivizumab. The present multi-center prospective study was conducted to clarify the genetic characteristics and variations in antigenic determinants of the F protein of the prevalent genotypes present in the mainland of China.

## Results

### The samples

In this study, a total of 553 (23.65%) RSV positive samples were identified from 2338 enrolled patients between November 2014 and June 2016 from 10 hospitals located in Beijing, Chongqing, Jilin, Hebei, Ningxia, Zhejiang, and Guangdong provinces in China. The ages of these patients ranged from 1 month to 8.5 years with a median age of 1 year. The male-to-female ratio was 2:1 (369 boys and 184 girls). For these 553 RSV positive samples, 366 (66.18%) were identified as RSV genotype A (RSV A), 176 (31.83%) were identified as RSV B, and 11 (1.99%) were identified as both RSV A and B). A total of 252 complete *F* gene sequences were obtained from 553 RSV positive samples, while the complete *F* gene sequences of 301 samples were not available because of poor sequence data quality.

### Phylogenetic analysis

A total of 394 RSV *F* gene complete sequences were analyzed in this study, including 252 complete *F* gene sequences obtained (183 RSV A and 69 RSV B) in this study, and 142 reference sequences (including Long, CH18537, and 140 Chinese RSV *F* sequences) available from GenBank. Except the prototype sequences of Long and CH18537 strain, pairwise nucleotide (amino acid) sequences identities were 94.3%–100% (96.1%–100%) among 300 RSV A; 96.5%–100% (97.9%–100%) among 92 RSV B; and 81.2%–82.7% (88.3%–90.9%) between groups A and B. Compared with the prototype strains Long (for RSV A) and CH18537 (RSV B), the mean distances were 0.049 for RSV A, and 0.034 for RSV B, respectively. The P-distance between RSV B and the prototype Long strain was 0.204.

**Figure 1:**
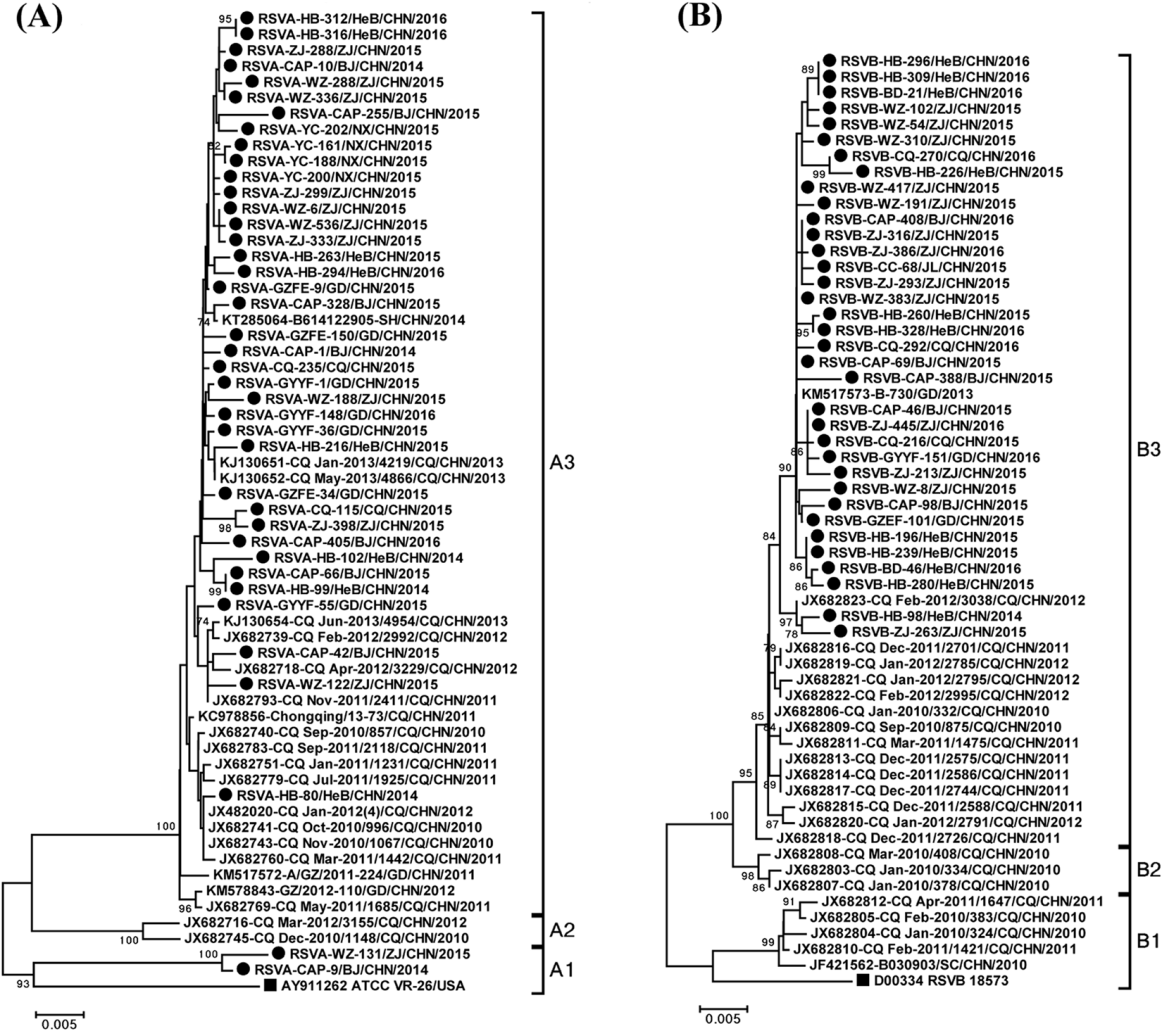
The phylogenetic dendrogram based on the alignments of the 120 representative RSV *F* gene complete sequences was constructed with MEGA5.03 software. Panels (A and B) are detailed phylogenetic trees of the RSV A and RSV B taxa analyzed, respectively. The neighbour-joining method was used to infer the topology. The percentage of bootstrap (percentage of 1000 pseudoreplicate datasets) replicates supporting the trees are indicated at the nodes (for clarity, only values over 70% are shown). Squares indicate the prototypes RSV A Long and RSV B CH18537 strains. Filled circles indicate the strains from this study during 2014–2016. The scale bars represent substitutions per base pair per the indicated horizontal distance.

All the Chinese RSV A strains could be stratified into A1, A2 and A3 branches. The mean genetic distances were 0.048, 0.049 and 0.035 between A1 and A2, A1 and A3, A2 and A3 respectively. RSV B strains could also be divided into three branches, with low genetic distance (0.027 between B1 and B2, 0.029 between B1 and B3, 0.012 between B2 and B3). Apart from the strains collected during 2010–2013, most of the strains isolated between 2014 and 2016 belonged to the A3 and B3 branches (Fig. 1).

### Variation analysis

There were 83 nucleotide changes in RSV A and 59 nucleotide changes in RSV B fusion protein genes, and 298 nucleotide differences were observed between RSV A and B. All nucleotide mutations were base substitutions, the majority of which resulted in synonymous amino acid changes. The amino acid of the F proteins were relatively conserved. In contrast, there were 15 and 18 significant amino acid changes in RSV A and B, compared with their respective reference strains, while 56 amino acid differences were found between the two groups.

Variations at the antigenic site areas were analyzed (Table 1). For RSV A, amino acid changes were found at: antigenic site I in AA residue 384 (V → I, 99.3%); antigenic site p27 in amino residues 122 (T → A, 4.3%), 124 (K → N, 100%) and 125 (T → N, 0.6%). For RSV B, amino acid changes were found at: antigenic site p27 in AA residues 116 (N → D, 3.3%) and 125 (L → P, 3.3%); antigenic site α2α3β3β4 in AA residues 172 (L → Q, 91.3%) and 173 (S → L, 73.9%); antigenic site MPE8 in AA residues 45 (F → L, 94.6%). The change of 276 (N → S) on palivizumab binding site of antigenic site II was observed in 98.0% RSV A and all the RSV B strains. The change at antigenic site α2α3β3β4 in AA residue 173 (S → L, 73.9%) was only found in the RSV B strains during 2015–2016. For the AA residue 202 of antigenic site ∅, all sequences except CH18537 were glutamine.

**Table 1:** Amino acid changes in antigenic sites and Cytotoxic T lymphocyte (CTL) epitopes of the fusion gene of RSV A (*n* = 300) and RSV B (*n* = 92) comparing with the prototype Long and CH18537 strains.

Antigenic Site	AA	RSV A & Long		RSV B & Long		RSV B & CH18537		Between RSV A & B	
		V^a^	F (%)	V	F (%)	V	F (%)	V	F (%)
**I**	380–400	—	—	N380S	100			N380S	100
		V384I	99.3	V384T	100			V, I384T	100
		—	—	P389S	100			P389S	100
II	254–277	N276S	98.0	N276S	100			N276S	2.0
III	422–438	—	—	—	—				
∅	62–69, 196–210	—	—	N67T	100			N67T	100
		—	—	D200N	100			D200N	100
				K201N, S	97.8, 2.2			K201N, S	100
						R202Q	100		
				K209Q	91.3			K209Q	91.3
p27	109–136	—	—	L111A	100			L111A	100
		—	—	R113Q	100			R113Q	100
				F114Y	100			F114Y	100
				N116D	3.3^b^	N116D	3.3^b^	N116D	3.3
				L119I	100			L119I	100
				N121T	100			N121T	100
		T122A	4.3					A122T	4.3
		K124N	100	K124N	100				
		T125N	0.6	T125L, P	96.7, 3.3	L125P	3.3	T, N125L, P	100
				T128S	100			T128S	100
				L129I	100			L129I	100
α2α3β3β4(AM14)	148–194			S169N	100			S169N	100
				L172Q	91.3	L172Q	91.3	L172Q	91.3
				S173L	73.9^b^	S173L	73.9^b^	S173L	73.9^b^
MPE8	44–50, 305–310			L45F	5.4	F45L	94.6	L45F	5.4
				L305I	100			L305I	100
HLA-A*01	109–118			L111A	100			L111A	100
				R113Q	100			R113Q	100
				F114Y	100			F114Y	100
				N116D	3.3^b^	N116D	3.3^b^	N116D	3.3^b^
HLA-B*57	106–114			L111A	100			L111A	100
				R113Q	100			R113Q	100
				F114Y	100			F114Y	100
HLA-Cw*12	542–550			V544I	100			V544I	100
		L547F	1.3	L547M	2.2	L547M	2.2	F547M	1.5
HLA-A*0201	10–18, 191–199, 250–258, 533–544	A10T	2.3					T10A	2.3
				T12F	100			T12F	100
				T13L	100			T13L	100
				I14T	100			I14T	100
						L15F	3.3^b^	L15F	3.3^b^
				A17I	100	V17I	100	A17I	100
		V18I	98.3	V18N	100			V, I 18N	100
				I537V	100			I537V	100
		S540A	98.0					A540S	98.0
				V544I	98.9			V544I	98.9

Notes. ^a^A position B, the amino acid of this site have a change from A to B; ^b^All from 2015–16.

Abbreviations: AA, amino acids; F, frequency; V, variation.

Mutations were also observed in the human histocompatibility leukocyte antigen (HLA) -restricted CTL epitopes. For RSV A, two amino acid residue substitutions were observed in over 98% of the strains in the HLA-A*0201 epitope, while the amino residue mutations 10 (A → T) in the HLA-A*0201 epitope, and 547 (L → F) in the HLA-Cw*12 epitope, were only found in 7 and 4 of the RSV A strains, respectively. For RSV B, comparing with the CH18537 strain, one mutation 17 (V → I) in HLA-A*0201 was observed among all the Chinese RSV B strains. The AA residue 15 (L → F) substitution in HLA-A*0201 and the 116 (N → D) in HLA-A*01 restricted epitopes were only observed in three strains between 2015 and 2016. The AA residue 547 (L → M) substitution in HLA-Cw*12 restricted epitopes was only observed in two strains.

## Discussion

The F protein of RSV is the significant candidate for RSV vaccine and antibody development. Although it is relatively conserved, many variations were found in *F* gene among different clinical strains, especially between RSV subtypes. Few data about the molecular epidemiology of RSV *F* gene is available in China. In this multi-center prospective study, 2338 patients were enrolled from 7 provinces of China during December 2014–June 2016. A total of 553 patients with RSV infection were identified. RSV A was the predominant subtype which was detected from 366 (66.18%) samples, while the RSV B strains (176, 31.83%) detection was slightly lower.

The RSV *F* complete gene was amplified and sequenced from 252 clinical samples. All Chinese RSV *F* sequences after 2010 available from GenBank were also analyzed in this study. The sequence identities within Chinese RSV A and B were more than 94.3% and 96.5% at the nucleotide level, more than 96.1% and 97.9% at the AA level, respectively. The sequence identities, including nucleotide and amino acid level, were lower than that reported by Xia *et al*.^7^. It might be due to the longer time and wider geographical distributions. The phylogenetic analyses showed that the RSV A strains from different regions of China between 2014 and 2016 were slightly clustered into the same branches, while the same temporal pattern of clusters of isolates was observed in RSV B.

There were a few AA changes in the antigenic sites of RSVA and RSVB by comparing with their historical Long and CH18537 strains, respectively. These changes contain the substitutions of basic/acidic AA (K124N, R202Q, N116D), hydrophilic/hydrophobic AA (L172Q, S173L), and replacement of heterocyclic AA (L125P). These changes might alter the secondary structure of the protein, which are much more likely to change the antigenic appearance. However, further study is required for identifying the contributions of these non-synonymous AA changes. RSV F protein has metastable and stable states, which corresponds to the prefusion and postfusion respectively. The prefusion F protein on the surface of the virus plays the function of driving membrane fusion between the viral envelope and the host cell. The prefusion F protein undergoes a dramatic conformational change at the initiation of fusion, which results a very stable postfusion form^13^. RSV neutralization activity in human sera is primarily directed against the antigenic sites on the prefusion F protein^14^. Comparing with the historical CH18537 strain, the antigenic sites of RSV B prefusion F protein (∅, MPE-8, α2α3β3β4 (AM14), and p27) were relatively much more variable than the postfusion F protein. The amino acid of the antigenic sites between RSV A and B were high variable. There were 23 amino acid changes between RSV A and B. These results may suggest that a monovalent vaccine based on the F protein of RSV A may not provide enough protection against infections caused by RSV B. This will be an important consideration for the development of the therapeutic monoclonal antibody or small molecular drugs.

The p27 is cleaved during the infection of cells and internalization of the virus. The cleavage of p27 is an important event to enable full infection of the virus^15^, and is also a proven significant antigenic site^16^. Indeed, the p27 was the most variable antigenic site between RSV A and –B in this study. However, within each group, RSV A and RSV B are relatively conserved compared with their respective reference strains. These results suggest that the p27 antigenic site is responsible for group specificity, which is consistent with previous study^8^.

It is interesting that the change at antigenic site α2α3β3β4 in AA residue 173 (S → L) was only found in 97.1% (67/69) of RSV B strains in China during 2015–2016. This was a new antigenic site change in clinical strains from China, however it was not found in RSV B before 2014 or in RSV A strains. This is not a resistance mutation under pressure, since no drugs or antibodies targeting this antigenic site was used. It might suggest that the change is due to the antigenic drift of the viruses and has become the predominant strain. Few data on antigenic site α2α3β3β4 was available. The potential effect of this change needs further study.

Palivizumab is the only licensed monoclonal antibody for the prevention of severe RSV infection. The binding site of palivizumab is located on the antigenic sites II, which existed in both the prefusion and postfusion forms of F protein. There were many reports about the change of 276 (N → S), which was proved to be a non-resistance mutation to palivizumab^7,17^. The change of 276 (N → S) was also observed in 98.0% RSV A and all the RSV B strains in this study. Consistent with the report of Xia *et al*.^7^, the percentage of strains with 276 (N → S) substitution in the F protein is increasing, accounted for 99% of RSV after 2014. The residues 262, 272, 275 are very important sites at which mutations could confer resistance to palivizumab^17^. No other change was observed in this study except the 276 (N → S) substitution. No resistance-associated mutations of natural polymorphisms or selection pressure to palivizumab were found in the RSV strains of China.

The amino acid variations at the CTL epitopes were also significant for RSV vaccine design and to understand the mechanism the virus uses to escape immunosurveillance^11^. The CTL epitopes were conserved within the groups in this study. Almost all the variations constituted inter-group differences, except for residues 18 (V → I) and 540 (S → A) in RSV A, and 17 (V → I) in RSV B. The AA residue 15 (L → F) substitution in HLA-A*0201 and the 116 (N → D) in HLA-A*01 restricted epitopes were first observed in three strains between 2015 and 2016. Continuous monitoring is needed to investigate the development of these mutations; and further studies are needed to illustrate the effect of these variations on recognition of CTL cells.

In conclusion, this is the first multi-center molecular epidemiologic study of the RSV *F* gene in China. The result revealed that the RSV *F* genes share relatively high identity in China. However, genetic variations temporally were also observed. The alterations of 276 (N → S) were observed in the majority of RSV A strains. The genetic variations of 173 (S → L) at antigenic site α2α3β3β4 and 15(L → F), 116 (N → D) at CTL epitopes were first identified in the RSV B strains during 2015–16. The F protein of RSV in the mainland of China is relatively conserved and remains the potential candidate target for vaccine and drug development.

## Methods

### Ethics statement

This study was approved by the Medical Ethics Committee of the Beijing Children’s Hospital affiliated to Capital Medical University, and informed consent was obtained. All procedures were conducted according to the guidelines.

### Sample collection

Between November 2014 and June 2016, RSV positive respiratory samples were collected from patients hospitalized with community-acquired pneumonia (CAP) in a multi-centers study that involved 10 hospitals of 7 provinces or municipalities (Beijing, Chongqing, Jilin, Hebei, Ningxia, Zhejiang, and Guangdong) in China. Eligible participants were younger than 18 years. Nasopharyngeal aspirates or throat swabs were collected on admission. The specimens were frozen at −80 °C until tested by a multiplex PCR/DNA hybridization assay that detect RSV A, RSV B, and 16 other respiratory viruses (xTAG RVP Kit; Luminex, Toronto, Ontario, Canada).

### RNA extraction and sequence determination for RSV *F* gene

Total RNA from RSV positive clinical samples were extracted by using QIAamp MinElute Virus Spin Kit (Qiagen, Hilden, Germany) according to the manufacturer’s instructions. The complete *F* gene sequences were obtained by segmented amplification and sequencing. Two fragments were amplified by SuperScript™ III One-Step RT-PCR System (Invitrogen, Carlsbad CA, USA, cat: 12574018), respectively. The primer pairs A-F1/R1 (A-F1: CTCAAGTCTCCACAACATCCGA; A-R1: GGGAAGAAAGATACTGATCCTGCAT) and F2/R2 (F2: ATGCCTATAACAAATGATCAGAAAAAGTT; R2: GCAATGACCTCGAATTTCAAATT) were used to amplify and sequence the nucleotides of the *F* gene of RSV A samples, while the primer pairs B-F1/R1 (B-F1: CACAAACACCCACAGCATCCGA; B-R1: GGAAAGAAGGATACTGATCCTGCAT) and F2/R2 were used to amplify and sequence the nucleotides of the *F* gene of RSV B samples. The sequences were edited and assembled by using Sequencher software version 5.0 (Gene Codes, Ann Arbor, MI, USA). The sequences generated in this study were submitted to GenBank with the accession numbers from MF978510 to MF978761.

### Other RSV *F* gene sequences used in this study

All (140) Chinese RSV *F* gene reference sequences (117 RSV A and 23 RSV B) from Jan 2010 to June 2017, as well as the prototype sequences of RSV A and B genotypes were downloaded from GenBank. Information of the sequences downloaded from GenBank is shown in the Supplementary Table 1.

### Phylogenetic and genetic diversity analysis

Multiple sequence alignments, phylogenetic analyses and pairwise distance were conducted and calculated by using MEGA 5.03 software (Sudhir Kumar, Arizona State University, Tempe, AZ, USA). The phylogenetic tree was generated with the neighbor-joining method and the Kimura 2-parameter model was chosen with pairwise deletion treatment for the missing and gaps data. The reliability of phylogenetic inference was estimated by using the bootstrap method with 1000 replicates, while only the values over 70% are shown. The sequences identities and genetic diversity of the complete *F* gene were determined with BioEdit software.

## Electronic supplementary material

Table S1
