# Supplementary material for: Genetic variations in the fusion protein of respiratory syncytial virus isolated from children hospitalized with community-acquired pneumonia in China
Source: Sci Rep. 2018 Mar 14;8:4491. doi: 10.1038/s41598-018-22826-4 (PMC5852162; doi:10.1038/s41598-018-22826-4)
Supplement: Supplementary file 1 — Table S1 [file 41598_2018_22826_MOESM1_ESM.doc]

**Genetic variations in the fusion protein of** **respiratory syncytial virus isolated from children hospitalized with community-acquired pneumonia in China**

Xiangpeng Chen^1^, Baoping Xu^2^, Jiayun Guo^1^, Changchong Li^3^, Shuhua An^4^, Yunlian Zhou^5^, Aihuan Chen^6^, Li Deng^7^, Zhou Fu^8^, Yun Zhu^1^, Chunyan Liu^1^, Lili Xu^1^, Wei Wang^1^, Kunling Shen^2^, and Zhengde Xie^1^

^1^ Key Laboratory of Major Diseases in Children, Ministry of Education, National Clinical Research Center for Respiratory Diseases, Beijing Key Laboratory of Pediatric Respiratory Infection Diseases, Beijing Pediatric Research Institute, Beijing Children’s Hospital, Capital Medical University, National Center for Children's Health, Beijing 100045, PR China.

^2^ Beijing Children’s Hospital, Capital Medical University, National Center for Children's Health, Beijing 10045, PR China.

^3^ The 2nd Affiliated Hospital and Yuying Children's Hospital of Wenzhou Medical University

^4^ Children’s Hospital of Hebei Province

^5^ The Children's Hospital-Zhejiang University School of Medical

^6^ The First Affiliated Hospital of Guangzhou Medical University

^7^ Guangzhou Women and Children's Medical Center

^8^ Children's Hospital of Chongqing Medical University

Xiangpeng Chen, Baoping Xu and Jiayun Guo contributed equally to this work.

Correspondence and requests for materials should be addressed to Z.X. (email: xiezhengde@bch.com.cn) or K.S. (email: kunlingshen1717@163.com)

Table S1. List of the 142 RSV complete *F* gene sequences downloaded from GenBank.

| Isolates | GenBank Accession Number | The place of isolation | The year of isolation | Subtype |
| --- | --- | --- | --- | --- |
| B614122905 | KT285064 | Shanghai | 2014 | A |
| A/GZ/12-110 | KM578843 | Guangdong | 2012 | A |
| A/GZ/11-224 | KM517572 | Guangdong | 2011 | A |
| CQ_Nov-2012/3998 | KJ130649 | Chongqing | 2012 | A |
| CQ_Dec-2012/4078 | KJ130650 | Chongqing | 2012 | A |
| CQ_Dec-2012/4219 | KJ130651 | Chongqing | 2013 | A |
| CQ_May-2013/4866 | KJ130652 | Chongqing | 2013 | A |
| CQ_May-2013/4945 | KJ130653 | Chongqing | 2013 | A |
| CQ_Jun-2013/4954 | KJ130654 | Chongqing | 2013 | A |
| CQ_Jun-2013/13-73 | KC978856 | Chongqing | 2011 | A |
| CQ_Mar-2012/3163 | JX682715 | Chongqing | 2012 | A |
| CQ_Mar-2012/3155 | JX682716 | Chongqing | 2012 | A |
| CQ_Mar-2012/3174 | JX682717 | Chongqing | 2012 | A |
| CQ_Apr-2012/3229 | JX682718 | Chongqing | 2012 | A |
| CQ_Oct-2011/2251 | JX682719 | Chongqing | 2011 | A |
| CQ_Oct-2011/2310 | JX682720 | Chongqing | 2011 | A |
| CQ_Oct-2011/2315 | JX682721 | Chongqing | 2011 | A |
| CQ_Oct-2011/2319 | JX682722 | Chongqing | 2011 | A |
| CQ_Nov-2011/2348 | JX682723 | Chongqing | 2011 | A |
| CQ_Nov-2011/2353 | JX682724 | Chongqing | 2011 | A |
| CQ_Nov-2011/2385 | JX682725 | Chongqing | 2011 | A |
| CQ_Dec-2011/2580 | JX682726 | Chongqing | 2011 | A |
| CQ_Dec-2011/2587 | JX682727 | Chongqing | 2011 | A |
| CQ_Dec-2011/2645 | JX682728 | Chongqing | 2011 | A |
| CQ_Dec-2011/2599 | JX682729 | Chongqing | 2011 | A |
| CQ_Dec-2011/2659 | JX682730 | Chongqing | 2011 | A |
| CQ_Dec-2011/2678 | JX682731 | Chongqing | 2011 | A |
| CQ_Jan-2012/2788 | JX682732 | Chongqing | 2012 | A |
| CQ_Jan-2012/2792 | JX682733 | Chongqing | 2012 | A |
| CQ_Jan-2012/2848 | JX682734 | Chongqing | 2012 | A |
| CQ_Jan-2012/2881 | JX682735 | Chongqing | 2012 | A |
| CQ_Jan-2012/2904 | JX682736 | Chongqing | 2012 | A |
| CQ_Jan-2012/2907 | JX682737 | Chongqing | 2012 | A |
| CQ_Jan-2012/2947 | JX682738 | Chongqing | 2012 | A |
| CQ_Feb-2012/2992 | JX682739 | Chongqing | 2012 | A |
| CQ_Sep-2012/857 | JX682740 | Chongqing | 2010 | A |
| CQ_Oct-2012/996 | JX682741 | Chongqing | 2010 | A |
| CQ_Oct-2012/1004 | JX682742 | Chongqing | 2010 | A |
| CQ_Nov-2010/1067 | JX682743 | Chongqing | 2010 | A |
| CQ_Dec-2010/1146 | JX682744 | Chongqing | 2010 | A |
| CQ_Dec-2010/1148 | JX682745 | Chongqing | 2010 | A |
| CQ_Dec-2010/1178 | JX682746 | Chongqing | 2010 | A |
| CQ_Dec-2010/1190 | JX682747 | Chongqing | 2010 | A |
| CQ_Dec-2010/1199 | JX682748 | Chongqing | 2010 | A |
| CQ_Dec-2010/1200 | JX682749 | Chongqing | 2010 | A |
| CQ_Dec-2010/1213 | JX682750 | Chongqing | 2010 | A |
| CQ_Jan-2011/1231 | JX682751 | Chongqing | 2011 | A |
| CQ_Jan-2011/1275 | JX682752 | Chongqing | 2011 | A |
| CQ_Jan-2011/1282 | JX682753 | Chongqing | 2011 | A |
| CQ_Feb-2011/1366 | JX682754 | Chongqing | 2011 | A |
| CQ_Feb-2011/1372 | JX682755 | Chongqing | 2011 | A |
| CQ_Feb-2011/1373 | JX682756 | Chongqing | 2011 | A |
| CQ_Feb-2011/1399 | JX682757 | Chongqing | 2011 | A |
| CQ_Mar-2011/1434 | JX682758 | Chongqing | 2011 | A |
| CQ_Mar-2011/1437 | JX682759 | Chongqing | 2011 | A |
| CQ_Mar-2011/1442 | JX682760 | Chongqing | 2011 | A |
| CQ_Mar-2011/1446 | JX682761 | Chongqing | 2011 | A |
| CQ_Mar-2011/1455 | JX682762 | Chongqing | 2011 | A |
| CQ_Mar-2011/1465 | JX682763 | Chongqing | 2011 | A |
| CQ_Mar-2011/1470 | JX682764 | Chongqing | 2011 | A |
| CQ_Mar-2011/1507 | JX682765 | Chongqing | 2011 | A |
| CQ_Apr-2011/1555 | JX682766 | Chongqing | 2011 | A |
| CQ_Apr-2011/1642 | JX682767 | Chongqing | 2011 | A |
| CQ_Apr-2011/1643 | JX682768 | Chongqing | 2011 | A |
| CQ_May-2011/1685 | JX682769 | Chongqing | 2011 | A |
| CQ_May-2011/1712 | JX682770 | Chongqing | 2011 | A |
| CQ_May-2011/1716 | JX682771 | Chongqing | 2011 | A |
| CQ_May-2011/1748 | JX682772 | Chongqing | 2011 | A |
| CQ_May-2011/1762 | JX682773 | Chongqing | 2011 | A |
| CQ_Jun-2011/1795 | JX682774 | Chongqing | 2011 | A |
| CQ_Jun-2011/1827 | JX682775 | Chongqing | 2011 | A |
| CQ_Jun-2011/1842 | JX682776 | Chongqing | 2011 | A |
| CQ_Jun-2011/1868 | JX682777 | Chongqing | 2011 | A |
| CQ_Jul-2011/1920 | JX682778 | Chongqing | 2011 | A |
| CQ_Jul-2011/1925 | JX682779 | Chongqing | 2011 | A |
| CQ_Jul-2011/1927 | JX682780 | Chongqing | 2011 | A |
| CQ_Jul-2011/1940 | JX682781 | Chongqing | 2011 | A |
| CQ_Sep-2011/2111 | JX682782 | Chongqing | 2011 | A |
| CQ_Sep-2011/2118 | JX682783 | Chongqing | 2011 | A |
| CQ_Sep-2011/2147 | JX682784 | Chongqing | 2011 | A |
| CQ_Oct-2011/2208 | JX682785 | Chongqing | 2011 | A |
| CQ_Oct-2011/2219 | JX682786 | Chongqing | 2011 | A |
| CQ_Oct-2011/2237 | JX682787 | Chongqing | 2011 | A |
| CQ_Oct-2011/2241 | JX682788 | Chongqing | 2011 | A |
| CQ_Oct-2011/2247 | JX682789 | Chongqing | 2011 | A |
| CQ_Nov-2011/2362 | JX682790 | Chongqing | 2011 | A |
| CQ_Nov-2011/2379 | JX682791 | Chongqing | 2011 | A |
| CQ_Nov-2011/2384 | JX682792 | Chongqing | 2011 | A |
| CQ_Nov-2011/2411 | JX682793 | Chongqing | 2011 | A |
| CQ_Dec-2011/2624 | JX682794 | Chongqing | 2011 | A |
| CQ_Dec-2011/2626 | JX682795 | Chongqing | 2011 | A |
| CQ_Dec-2011/2627 | JX682796 | Chongqing | 2011 | A |
| CQ_Dec-2011/2628 | JX682797 | Chongqing | 2011 | A |
| CQ_Dec-2011/2639 | JX682798 | Chongqing | 2011 | A |
| CQ_Dec-2011/2640 | JX682799 | Chongqing | 2011 | A |
| CQ_Dec-2011/2641 | JX682800 | Chongqing | 2011 | A |
| CQ_Jan-2012(8) | JX482019 | Chongqing | 2012 | A |
| CQ_Jan-2012(9) | JX482018 | Chongqing | 2012 | A |
| CQ_Jan-2012(4) | JX482020 | Chongqing | 2012 | A |
| CQ_Jan-2012(19) | JX482021 | Chongqing | 2012 | A |
| CQ_Jan-2012(18) | JX482022 | Chongqing | 2012 | A |
| CQ_Jan-2012(15) | JX482023 | Chongqing | 2012 | A |
| CQ_Jan-2012(14) | JX482024 | Chongqing | 2012 | A |
| CQ_Jan-2012(13) | JX482025 | Chongqing | 2012 | A |
| CQ_Jan-2012(12) | JX482026 | Chongqing | 2012 | A |
| CQ_Jan-2012(11) | JX482027 | Chongqing | 2012 | A |
| CQ_Jan-2012(10) | JX482028 | Chongqing | 2012 | A |
| CQ_Jan-2012(1) | JX482029 | Chongqing | 2012 | A |
| CQ_Jan-2012(27) | JX482030 | Chongqing | 2011 | A |
| CQ_Jan-2012(25) | JX482031 | Chongqing | 2011 | A |
| CQ_Jan-2012(23) | JX482032 | Chongqing | 2011 | A |
| CQ_Jan-2012(22) | JX482033 | Chongqing | 2011 | A |
| CQ_Jan-2012(21) | JX482034 | Chongqing | 2011 | A |
| CQ_Jan-2012(18) | JX482035 | Chongqing | 2011 | A |
| CQ_Jan-2012(17) | JX48203 | Chongqing | 2011 | A |
| CQ_Jan-2012(16) | JX482037 | Chongqing | 2011 | A |
| CQ_Jan-2012(15) | JX482038 | Chongqing | 2011 | A |
| B/GZ/2013-730 | KM517573 | Guangdong | 2013 | B |
| CQ_Jan-2010/334 | JX682803 | Chongqing | 2010 | B |
| CQ_Jan-2010/324 | JX682804 | Chongqing | 2010 | B |
| CQ_ Feb-2010/383 | JX682805 | Chongqing | 2010 | B |
| CQ_Jan-2010/332 | JX682806 | Chongqing | 2010 | B |
| CQ_Jan-2010/378 | JX682807 | Chongqing | 2010 | B |
| CQ_ Mar-2010/408 | JX682808 | Chongqing | 2010 | B |
| CQ_Sep-2010/875 | JX682809 | Chongqing | 2011 | B |
| CQ_Feb-2010/1421 | JX682810 | Chongqing | 2011 | B |
| CQ_Mar-2011/1475 | JX682811 | Chongqing | 2011 | B |
| CQ_Apr-2011/1647 | JX682812 | Chongqing | 2011 | B |
| CQ_Dec-2011/2575 | JX682813 | Chongqing | 2011 | B |
| CQ_Dec-2011/2586 | JX682814 | Chongqing | 2011 | B |
| CQ_Dec-2011/2588 | JX682815 | Chongqing | 2011 | B |
| CQ_Dec-2011/2701 | JX682816 | Chongqing | 2011 | B |
| CQ_Dec-2011/2744 | JX682817 | Chongqing | 2011 | B |
| CQ_Dec-2011/2726 | JX682818 | Chongqing | 2011 | B |
| CQ_Jan-2012/2785 | JX682819 | Chongqing | 2012 | B |
| CQ_Jan-2012/2791 | JX682820 | Chongqing | 2012 | B |
| CQ_Jan-2012/2795 | JX682821 | Chongqing | 2012 | B |
| CQ_Feb-2012/2995 | JX682822 | Chongqing | 2012 | B |
| CQ_Feb-2012/3038 | JX682823 | Chongqing | 2012 | B |
| B030903 | JF421562 | Chongqing | 2010 | B |
| Long | AY911262 | USA | 1956 | A |
| CH-18537 | D00334 | USA | 1962 | B |
